# Insight into the Molecular Mechanism for the Discrepant Inhibition of Microcystins (MCLR, LA, LF, LW, LY) on Protein Phosphatase 2A

**DOI:** 10.3390/toxins14060390

**Published:** 2022-06-03

**Authors:** Yixue Xu, Jiyuan Cui, Huiqun Yu, Wansong Zong

**Affiliations:** College of Geography and Environment, Shandong Normal University, Jinan 250061, China; xuyixue2020@163.com (Y.X.); cjy987698461@163.com (J.C.); yu1375606933@163.com (H.Y.)

**Keywords:** microcystins, protein phosphatase 2A, inhibition mechanism, homology modeling, molecule simulation

## Abstract

Microcystins (MCs) exhibit diversified inhibition effects on protein phosphatases (PPs) due to their structural differences. To fully evaluate the potential mechanism for the discrepant inhibition effects, the five most frequent MCs with varying residues at position Z^4^ were selected as the tested toxins. Their inhibition sequence on PP2A was detected as follows: MCLR > MCLW > MCLA > MCLF > MCLY. Combined with homology modeling and molecular docking technology, the major interaction parameters between the MCs and PP2A were obtained. The correlation analysis for the major interaction parameters and inhibition effects showed that the hydrophobicity of Z^4^ had an important influence on the interaction of the MCs to PP2A. The introduction of hydrophobic Z^4^ directly weakened hydrogen bonds Z^4^→Pro_213_ and Z^4^←Arg_214_, indirectly weakened hydrogen bonds Adda^5^←Asn_117_, Glu^6^←Arg_89_, and MeAsp^3^←Arg_89_, but indirectly enhanced ionic bonds Glu^6^←Arg_89_, Glu^6^-Mn_1_^2+^, and Glu^6^-Mn_2_^2+^. In this way, the combination of the MCs with PP2A was blocked, and thus, the interactions between PP2A and the Mn^2+^ ions (in the catalytic center) were further affected; metal bonds Asp_85_-Mn_1_^2+^ and Asp_85_-Mn_2_^2+^ were weakened, while metal bond His_241_-Mn_1_^2+^ was enhanced. As a result, the interactions in the catalytic center were inhibited to varying degrees, resulting in the reduced toxicity of MCs.

## 1. Introduction

Toxic microcystins (MCs), produced by blue-green cyanobacterial, pose a worldwide threat to humans and wildlife [1,2]. When orally ingested, MCs can be transported to the liver by organic anion transport proteins [3,4]. Within the hepatic cells, MCs are inclined to inhibit the activity of serine/threonine protein phosphatase 1 and 2A (PP1 and PP2A) [5,6]. In this way, the balance between protein phosphorylation and dephosphorylation is destroyed, and thus, the cytoskeleton of hepatic cells is disrupted [7,8].

MCs are a class of monocyclic heptapeptides that share a common structure of cyclo-(d-Ala^1^-L-X^2^-d-isoAsp^3^-l-Z^4^-Adda^5^-d-isoGlu^6^-Mdha^7^) [9]. Due to the variable amino acids at positions 2 and 4, multiple variants have been identified [1,10]. Among MC variants, the most widespread and toxic congener MCLR was widely studied [11]. In consideration of its environmental risk, the World Health Organization recommended a provisional guideline value for MCLR (1 μg/L) in drinking water [12]. Based on the crystal structure analysis for the complexes of MCLR-PP1 and MCLR-PP2A, a two-step inhibition process was certified: for the reversible step, the hydrophobic side chain of Adda^5^ is rapidly wrapped in the hydrophobic cage structures of PP1/PP2A; for the irreversible step, the C=C double bond of Mdha^7^ undergoes an electrophilic addition reaction with the nucleophilic sites (Cys residue) in PP1/PP2A [13,14,15]. Both two steps together lead to decreased catalytic activity and cell necrosis.

**Figure 1:**
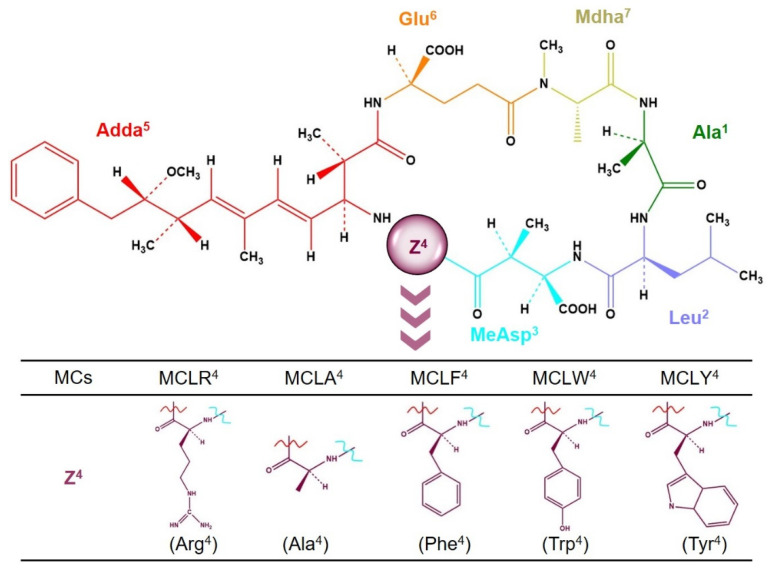
Chemical structures of MCs with varying amino acids at position Z^4^. Conditions: The blue and brown lines are connected to MeAsp^3^ and Adda^5^, respectively.

Compared with MCLR, other MC variants also have cyclic peptide structures and identical Adda^5^/Mdha^7^ residues (Figure 1) [8,16]. The inhibition processes for other variants to PP1/PP2A also should include the reversible and irreversible steps. Due to the structural differences of MC variants, their inhibition effects on PP1/PP2A exhibit diversification [17,18]. As information about the crystal structures of MCs-PP1/PP2A is limited, it is difficult to elucidate the molecular mechanism for the discrepant inhibition of MCs on PP1/PP2A. Homology modeling could evaluate the interactions between structural analogues and macromolecules, and it has been successively used to evaluate the interactions between drugs/contaminants and proteins [19,20,21].

Based on the homology modeling strategy, the interactions between MCs and PP1/PP2A can be simulated, and the molecular mechanism for the discrepant inhibition effects of MCs can be evaluated more in-depth. The five most frequent MCs with the changed Z^4^ residues (MCLR, LF, LA, LY, and LW) were selected as the typical variants, and their inhibition effects on PP2A were explored by a colorimetric protein phosphatase inhibition assay [22,23]. With the assistance of the molecular simulation, the models for MC-PP2A were constructed based on the crystal structure of the MCLR-PP2A complex. The major interaction parameters (such as combination energy changes, combination areas, related surface areas, and related chemical bonds) between typical MCs and PP2A were obtained by molecular docking. By analyzing the correlation between the inhibition data and the major interaction parameters, the key interactions and related sites were filtrated. Thus, the molecular mechanism for the discrepant inhibition of typical MCs on PP2A was clarified.

## 2. Results and Discussion

### 2.1. Evaluation of the Inhibition Effect of Typical MCs Targeted to PP2A

**Figure 2:**
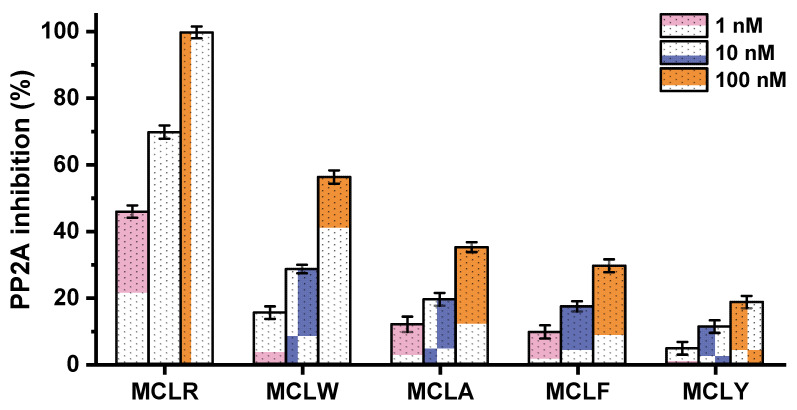
The inhibition effect of routinely detected MCs on PP2A at 1 nM, 10 nM, and 100 nM with its standard deviation (*n* = 3).

A traditional colorimetric PP inhibition assay was carried out to evaluate the inhibition effect of MCs on PP2A. As shown in Figure 2, all the MCs exhibited inhibition effects on PP2A and showed dose-effect relationships at 1 nM, 10 nM, and 100 nM. The inhibition sequence was MCLR > MCLW > MCLA > MCLF > MCLY. Among the variants, MCLR had a much higher inhibition effect than other MCs. By comparing the amino acid residues at position Z^4^, it was found that the electropositive Arginine (R) is more hydrophilic than the other four amino acids. When Arg^4^ was replaced by Trp^4^/Tyr^4^/Ala^4^/Phe^4^ (with increased hydrophobicity), the corresponding inhibition effect generally decreased. The lower inhibition of MCLY might be attributed to the electronegative OH in the side-chain of Tyr^4^. Unfortunately, the crystal structures for most MC-PP2A complexes (except for MCLR-PP2A) have not been prepared and solved. It would be difficult to elucidate the relationship between structural differences (changed Z^4^) and the discrepant inhibition of MCs on PP2A.

### 2.2. Filtration of the Major Interaction Parameters between MCs and PP2A Based on Homology Modeling

**Figure 3:**
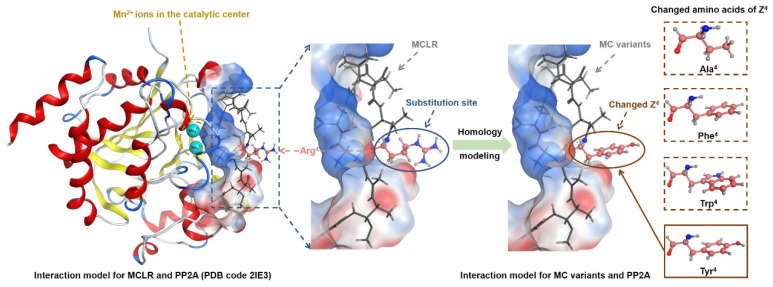
Illustration for the model construction of MC-PP2A complexes (with no PDB models) based on the homology modeling strategy.

The homology modeling strategy has been widely used to evaluate the interactions between structural analogues and proteins [24,25]. Based on this strategy, the interaction models for typical MCs and PP2A could be obtained by molecular simulation (Figure 3). The model for the MCLR-PP2A complex was revised from a crystal model in Protein Data Bank (PDB code 2IE3). The models for other MC-PP2A complexes were obtained by substituting the Arg^4^ residue in MCLR with Ala^4^, Phe^4^, Trp^4^, and Tyr^4^. With the help of molecular docking, the major interaction parameters for MC-PP2A complexes were obtained and are listed in Table S1. The major interaction parameters include the changed energies, combination areas, related surface areas, catalytic center exposure areas, the related hydrogen bonds, ionic bonds, and metal bonds. As PP2A was a type of metalloenzyme and regulated by two Mn^2+^ ions, the interaction parameters related to the Mn^2+^ ions were obtained simultaneously. Besides, the basic parameters (logP and logS) for typical MCs were also calculated.

### 2.3. Pearson Correlation Analysis for Inhibition Data and the Major Interaction Parameters

To evaluate the discrepant inhibition mechanism of MCs on PP2A, the correlation between inhibition data and the major interaction parameters was further evaluated by the Pearson correlation analysis. Regression analysis was not used to avoid deleting valid parameters associated with a few finite amino acid residues.

**Figure 4:**
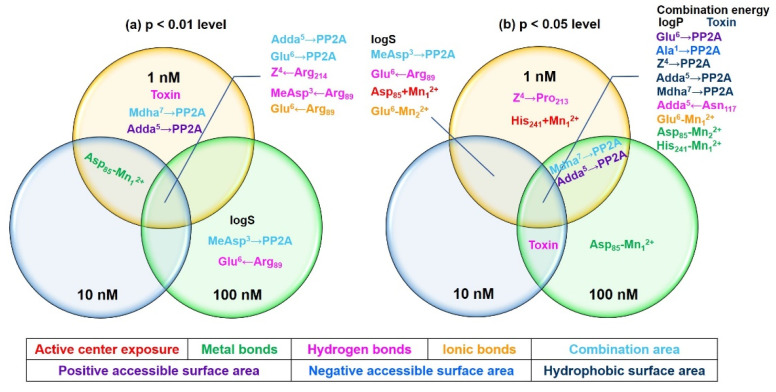
Venn diagrams of the important interaction parameters at the *p* < 0.01 level (**a**) and the *p* < 0.05 level (**b**). Different colors represent different kinds of interaction parameters. Condition: Toxin is different kinds of integral parameters.

Table 1 shows the major interaction parameters that exhibited diversified correlations with the inhibition effect of the MCs. To filtrate the important interaction parameters, Venn diagrams were drawn (Figure 4). At the *p* < 0.01 level, the combination areas for Adda^5^→PP2A and Glu^6^→PP2A, the hydrogen bonds for Z^4^←Arg_214_ and MeAsp^3^←Arg_89_, as well as the ionic bond for Glu^6^←Arg_89_ were significantly correlated with the inhibition effect at the three test concentrations. The metal bond for Asp_85_-Mn_1_^2+^ was significantly correlated with the inhibition effect at 1 nM and 10 nM. Total hydrogen bonds, the combination area for Mdha^7^→PP2A, and the positive accessible surface area for Adda^5^→PP2A were significantly correlated with the inhibition effect at 1 nM. LogS, the combination area for MeAsp^3^→PP2A, and the hydrogen bond for Glu^6^←Arg_89_ were significantly correlated with the inhibition effect at 100 nM. At the *p* < 0.05 level, combination energy change, logP, the hydrophobic surface area for toxin→PP2A, the positive accessible surface area for Glu^6^→PP2A, the negative accessible surface area for Ala^1^→PP2A, the hydrophobic surface areas for Adda^5^→PP2A/Mdha^7^→PP2A, the hydrogen bond for Adda^5^←Asn_117_, the ionic bond for Glu^6^-Mn_1_^2+^, the metal bonds for Asp_85_-Mn_2_^2+^, His_241_-Mn_1_^2+^, and combination energy change were significantly correlated with the inhibition effect at three test concentrations. LogS, the combination area for MeAsp^3^→PP2A, the negative accessible surface area for MeAsp^3^→PP2A, the hydrogen bond for Glu^6^←Arg_89_, the catalytic center exposure area for Asp_85_ + Mn_1_^2+^, and the ionic bond for Glu^6^-Mn_2_^2+^ were significantly correlated with the inhibition effect at 1 nM and 10 nM. The combination area for Mdha^7^→PP2A and the positive accessible surface area for Adda^5^→PP2A were significantly correlated with the inhibition effect at 1 nM and 100 nM. Total hydrogen bonds were significantly correlated with the inhibition effect at 10 nM and 100 nM. The hydrogen bond for Z^4^→Pro_213_ and the catalytic center exposure area for His_241_ + Mn_1_^2+^ were significantly correlated with the inhibition effect at 1 nM. The metal bond for Asp_85_-Mn_1_^2+^ was significantly correlated with the inhibition effect at 100 nM. Obviously, the above interaction parameters (especially the parameters significantly correlated with the inhibition effect at two or three test concentrations) were important for the combination of the MCs to PP2A.

### 2.4. Molecular Mechanism Analysis of the Discrepant Inhibition of MCs on PP2A

**Figure 5:**
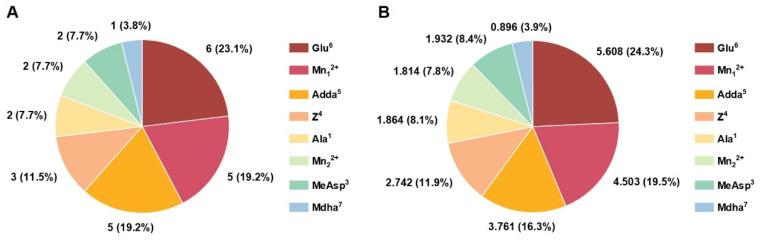
Pie charts for the statistical frequency (**A**) and the total |$\overset{–}{R}$| values (**B**) related to the key interaction sites. Conditions: $\overset{–}{R}$ is the average of Pearson correlations at three toxin concentrations.

According to the Pearson correlation analysis, the integral parameters such as total hydrogen bonds (|$\overset{–}{R}$| = 0.945), logS (|$\overset{–}{R}$| = 0.925), logP (|$\overset{–}{R}$| = 0.918), ASA-H for toxin→PP2A (|$\overset{–}{R}$| = 0.913), and combination energy change (|$\overset{–}{R}$| = 0.890) were highly correlated with the inhibition effect of the MCs. Obviously, logP, logS, and hydrophobic surface area (ASA-H) were associated with the hydrophobicity of MCs. When the hydrophilic Arg^4^ was substituted with Trp^4^/Ala^4^/Phe^4^/Tyr^4^, logP and hydrophobic surface areas (ASA-H) for MCs gradually increased, while logS showed a downward trend. In view of this, the hydrophobicity should have an important influence on the combination of MCs to PP2A; the increased hydrophobicity of Z^4^ would hinder the combination of MCs to PP2A by weakening the total hydrogen bonds (negative correlated with inhibition effect) and by acting on specific important interactions. Statistical frequency analysis of the key sites associated with the important interaction parameters (Figure 5A) showed that Glu^6^, Mn_1_^2+^, Adda^5^, Z^4^, Ala^1^, Mn_1_^2+^, MeAsp^3^, and Mdha^7^ participated in the interactions between MCs and PP2A in varying degrees. Combined with the statistical analysis for the total |$\overset{–}{R}$| values related to the above sites (Figure 5B), it could be found that the influence of Glu^6^, Mn_1_^2+^, and Adda^5^ was more significant than that of Z^4^ and other sites. Obviously, the hydrophobicity of Z^4^ mainly influenced the combination of MCs to PP2A in an indirect way.

**Figure 6:**
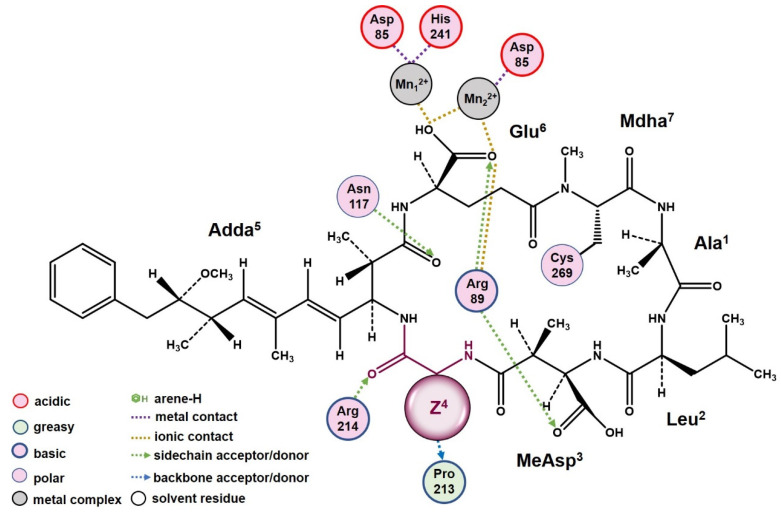
The 2D ligand-receptor interaction diagram for the combination of the MCs with PP2A.

A two-dimensional ligand-receptor interaction diagram for the combination of MCs with PP2A illustrated the key interactions, including hydrogen bonds MeAsp^3^←Arg_89_, Z^4^←Arg_214_, Z^4^→Pro_213_, Adda^5^←Asn_117_, and Glu^6^←Arg_89_, ionic bonds Glu^6^←Arg_89_, Glu^6^-Mn_1_^2+^, and Glu^6^-Mn_2_^2+^, and metal bonds Asp_85_-Mn_1_^2+^, Asp_85_-Mn_2_^2+^, and His_241_-Mn_1_^2+^ (Figure 6). The Pearson correlation analysis showed the hydrophobicity of Z^4^ had the most important influence on the interactions of the MCs to PP2A. According to the hydrophobic surface area analysis, with the increased hydrophobicity of Z^4^, the hydrophobic surface area between Z^4^ and PP2A increased. The hydrophobic combination of Z^4^ with PP2A directly weakened hydrogen bonds Z^4^←Arg_214_ and Z^4^→Pro_21__3_ to varying degrees. The hydrophobic combination of Z^4^ with PP2A could intervene the interactions between other residues of MCs and PP2A by weakening the hydrogen bonds Adda^5^←Asn_117_ (hydrophobic competition), Glu^6^←Arg_89_, MeAsp^3^←Arg_89_, and enhancing ionic bonds Glu^6^←Arg_89_, Glu^6^-Mn_1_^2+^, and Glu^6^-Mn_2_^2+^. Correspondingly, the combination areas of MeAsp^3^, Adda^5^, and Glu^6^ with PP2A all decreased. As a result, the combination of the MCs with PP2A was blocked to certain degrees. Subsequently, the interactions between PP2A and the Mn^2+^ ions in the catalytic center were further affected; the metal bonds Asp_85_-Mn_1_^2+^ and Asp_85_-Mn_2_^2+^ were weakened, while the metal bond His_241_-Mn_1_^2+^ was enhanced. As a result, there was an increase in the exposure area of the catalytic center (especially for Mn_1_^2+^ ion), resulting in lower inhibition effects of the MCs. 

## 3. Conclusions

To obtain a better understanding of the discrepant inhibition effect of the MCs on PP2A, the five most frequent MCs with the changed Z^4^ residues (MCLR, LF, LA, LY, and LW) were selected as the typical variants. A protein phosphatase inhibition assay showed that their inhibition effects on PP2A were in the sequence of MCLR > MCLW > MCLA > MCLF > MCLY. With the assistance of molecular modeling, the interaction models for MC-PP2A were constructed by homology modeling, and the major interaction parameters between MCs and PP2A were obtained by molecular docking. The Pearson correlation analysis for the major interaction parameters and inhibition data verified the important influence of the hydrophobicity of Z^4^ on MC toxicity. The increased hydrophobicity of Z^4^ directly promoted the hydrophobic combination of Z^4^ to PP2A and weakened the hydrogen bonds Z^4^←Arg_214_ and Z^4^→Pro_213_. The hydrophobic combination of Z^4^ with PP2A could intervene with the interactions between other residues of the MCs and PP2A by weakening the hydrogen bonds MeAsp^3^←Arg_89_, Adda^5^←Asn_117_, and Glu^6^←Arg_89_ and by enhancing the ionic bonds Glu^6^←Arg_89_, Glu^6^-Mn_1_^2+^ and Glu^6^-Mn_2_^2+^. The above key interactions between the MCs and PP2A further influenced the interactions between PP2A and the Mn^2+^ ions (the metal bonds Asp_85_-Mn_1_^2+^ and Asp_85_-Mn_2_^2+^ were weakened but the metal bond His_241_-Mn_1_^2+^ was enhanced). The eventual result was the increased exposure area of the catalytic center (especially for the Mn_1_^2+^ ion) and the lower inhibition effect on PP2A.

## 4. Materials and Methods

### 4.1. Materials

MCLR, MCLF, MCLA, MCLY, and MCLW were purchased from Sigma (Saint-Quentin Fallavier, France). PP2A was purchased from New England Biolabs Inc (Beverly, MA, USA). Bovine serum albumin, dithiothreitol, MnCl_2_, P-nitrobenzene disodium orthophosphate, sodium thiosulfate, tris(hydroxymethyl) aminomethane, and other reagents were purchased from Sinopharm (Shanghai, China).

### 4.2. PP2A Inhibition Assay

The biological toxicity of the typical MCs was evaluated by a colorimetric protein phosphatase inhibition assay modified by Zong et al. [22,23]. First, PP2A was diluted to 5 U/mL with a buffer solution (50 mM Tris-HCl pH 7.4, 2.0 mM dithiothreitol, 1 mM MnCl_2_) and 1.0 g/L bovine serum albumin. Then, 10 μL of PP2A and 100 μL of test samples were mixed in a 96-well polystyrene microplate. With gentle shaking, the microtiter plates were kept at 25.0 °C for 15 min, and 90 μL p-nitrophenyl disodium orthophosphate (5 mM) was added. After 1 h, the absorbances at 405 nm were measured in a microplate reader. The inhibition of the test samples on PP2A was calculated by the formula of I_PP2A_ (%) = (A_control_ − A_sample_)/A_control_ × 100%. A_control_ and A_sample_ were the absorbance of the reference sample (without PP2A) and the test sample at 405 nm, respectively.

### 4.3. Molecular Docking for the Interactions between MCs and PP2A

The molecular docking simulation was performed with MOE software (version 20.09, Cloud Scientific, Shanghai, China). The original model for the MCLR-PP2A complex was obtained from the Protein Data Bank (PDB code 2IE3). Hydrogen atoms and charges were supplemented to PP2A to obtain the revised model. Based on the homology modeling strategy, models for MCLA-PP2A, MCLF-PP2A, MCLY-PP2A, and MCLW-PP2A complexes were obtained by substituting Arg^4^ (in the revised model of MCLR-PP2A) with Ala^4^, Phe^4^, Trp^4^, and Tyr^4^, respectively [24,26]. All the models for MC-PP2A complexes were minimized for energy optimization. To ensure the comparability between MCs, “Template dock” mode was used (the options for “Placement” and “Refinement” were set to no change). The specific docking parameters were set as follows: amber 10 EHT, solvation r-field, temperature 25.0°C, pH 7.4, salinity 0.05 M. The major interaction parameters (combination energies, combination areas, related surface areas, catalytic center exposure areas, the hydrogen bonds/ionic bonds/metal bonds for major interaction sites) and basic property parameters (logP/logS) for the MCs were obtained to evaluate the interactions between the MCs and PP2A.

### 4.4. Statistical Analysis

The Pearson correlation analysis was used to analyze the correlation between the inhibition data and the major interaction parameters by IBM SPSS statistics (version 26.0, Chicago, IL, USA).

## Figures and Tables

**Table 1:** Pearson correlation analysis of the inhibition data and the major interaction parameters.

Correlation Analysis Data ^a^		Combination Energy (KJ/Mol)	Combination Area (Å^2^)							
			Total	Ala^1^→PP2A	Leu^2^→PP2A	MeAsp^3^→PP2A	Z^4^→PP2A	Adda^5^→PP2A	Glu^6^→PP2A	Mdha^7^→PP2A
1 nM ^b^	R (p)	−0.892 * (0.042)	0.346 (0.569)	−0.392 (0.514)	−0.341 (0.574)	0.902 * (0.037)	0.282 (0.646)	0.978 ** (0.004)	0.979 ** (0.004)	0.978 ** (0.004)
10 nM ^b^	R (p)	−0.890 * (0.043)	0.464 (0. 431)	−0.438 (0. 461)	−0.254 (0.680)	0.951 * (0.013)	0.413 (0.490)	0.994 ** (0.001)	0.996 ** (0.000)	0.956 * (0.011)
100 nM ^b^	R (p)	−0.888 * (0.044)	0.523 (0.365)	−0.538 (0.350)	−0.172 (0.782)	0.982 ** (0.003)	0.492 (0.400)	0.980 ** (0.003)	0.979 ** (0.004)	0.910 * (0.032)
Correlation Analysis Data ^a^		logP (o/w)	Positive accessible surface area (Å^2^)							
			Total	Ala^1^→PP2A	Leu^2^→PP2A	MeAsp^3^→PP2A	Z^4^→PP2A	Adda^5^→PP2A	Glu^6^→PP2A	Mdha^7^→PP2A
1 nM	R (p)	−0.929 * (0.023)	0.456 (0.440)	−0.765 (0.132)	−0.652 (0.233)	0.125 (0.841)	0.177 (0.775)	0.888 ** (0.044)	0.910 * (0.032)	−0.386 (0.521)
10 nM	R (p)	−0.936 * (0.019)	0.581 (0.305)	−0.712 (0.177)	−0.560 (0.326)	0.245 (0.691)	0.321 (0.598)	0.905 * (0.034)	0.922 * (0.026)	−0.410 (0.494)
100 nM	R (p)	−0.888 * (0.044)	0.660 (0.226)	−0.593 (0.292)	−0.419 (0.483)	0.345 (0.570)	0.424 (0.477)	0.900 * (0.037)	0.886 * (0.046)	−0.345 (0.569)
Correlation Analysis Data ^a^		logS	Negative accessible surface area (Å^2^)							
			Total	Ala^1^→PP2A	Leu^2^→PP2A	MeAsp^3^→PP2A	Z^4^→PP2A	Adda^5^→PP2A	Glu^6^→PP2A	Mdha^7^→PP2A
1 nM	R (p)	0.884 * (0.046)	0.388 (0.519)	0.882 * (0.048)	−0.548 (0.339)	0.394 (0.512)	0.466 (0.429)	0.378 (0.530)	0.474 (0.419)	−0.140 (0.822)
10 nM	R (p)	0.925 * (0.025)	0.512 (0. 378)	0.892 * (0.042)	−0.607 (0.278)	0.324 (0.595)	0.560 (0.326)	0.390 (0.516)	0.378 (0.531)	−0.225 (0.716)
100 nM	R (p)	0.966 ** (0.008)	0.585 (0.300)	0.915 * (0.029)	−0.707 (0.182)	0.179 (0.773)	0.583 (0.302)	0.429 (0.471)	0.303 (0.620)	−0.365 (0.546)
Correlation Analysis Data ^a^		Hydrophobic surface area (Å^2^)								
		Total	Ala^1^→PP2A	Leu^2^→PP2A	MeAsp^3^→PP2A	Z^4^→PP2A	Adda^5^→PP2A	Glu^6^→PP2A	Mdha^7^→PP2A	
1 nM	R (p)	−0.929 * (0.022)	−0.087 (0.889)	0.590 (0.295)	−0.763 (0.134)	−0.900 * (0.038)	0.924 * (0.025)	0.564 (0.322)	0.934 * (0.020)	
10 nM	R (p)	−0.920 * (0.027)	−0.033 (0.958)	0.607 (0.277)	−0.734 (0.158)	−0.938 * (0.018)	0.953 * (0.012)	0.645 (0.240)	0.923 * (0.026)	
100 nM	R (p)	−0.889 * (0.044)	0.063 (0.920)	0.546 (0.341)	−0.730 (0.161)	−0.932 * (0.021)	0.953 * (0.012)	0.757 (0.138)	0.891 * (0.042)	
Correlation Analysis Data ^a^		Hydrogen bonds (KJ/Mol)								
		Total	Z^4^→Pro_213_	Ala^1^←Arg_268_	Leu^2^←Arg_89_	MeAsp^3^←Arg_89_	Z^4^←Arg_214_	Adda^5^←His_118_	Glu^6^←Arg_89_	Mdha^7^←Arg_268_
1 nM	R (p)	−0.980 ** (0.003)	−0.893 * (0.041)	−0.244 (0.693)	−0.780 (0.119)	−0.977 ** (0.004)	−0.995 ** (0.000)	0.216 (0.727)	−0.880 * (0.049)	−0.020 (0.975)
10 nM	R (p)	−0.953 * (0.012)	−0.841 (0.074)	−0.372 (0.537)	−0.798 (0.106)	−0.993 ** (0.001)	−0.995 ** (0.000)	0.284 (0.643)	−0.938 * (0.018)	0.128 (0.837)
100 nM	R (p)	−0.901 * (0.037)	−0.764 (0.133)	−0.521 (0.368)	−0.777 (0.122)	−0.991 ** (0.001)	−0.971 ** (0.006)	0.319 (0.600)	−0.984 ** (0.002)	0.296 (0.628)
Correlation Analysis Data ^a^		Hydrogen bond	Metal bonds (KJ/Mol)							
		Adda^5^←Asn_117_	Total	Glu^6^-Mn_1_^2+^	Glu^6^-Mn_2_^2+^	Asp_57_-Mn_1_^2+^	Asp_57_-Mn_2_^2+^	Asp_85_-Mn_1_^2+^	Asp_85_-Mn_2_^2+^	His_241_-Mn_1_^2+^
1 nM	R (p)	−0.904 * (0.035)	−0.519 (0.371)	0.517 (0.372)	0.731 (0.161)	0.742 (0.151)	−0.850 (0.068)	−0.985 ** (0.002)	−0.935 * (0.020)	0.926 * (0.024)
10 nM	R (p)	−0.946 * (0.015)	−0.571 (0.314)	0.400 (0.505)	0.621 (0.264)	0.792 (0.110)	−0.796 (0.107)	−0.964 ** (0.008)	−0.937 * (0.019)	0.956 * (0.011)
100 nM	R (p)	−0.959 * (0.001)	−0.669 (0.217)	0.287 (0.640)	−0.480 (0.414)	0.773 (0.126)	−0.688 (0.199)	−0.904 * (0.035)	−0.894 * (0.041)	0.936 * (0.019)
Correlation Analysis Data ^a^		Ionic bonds (KJ/Mol)								
		Total	Leu^2^←Arg_89_	MeAsp^3^←Arg_89_	Glu^6^←Arg_89_	Glu^6^-Mn_1_^2+^	Glu^6^-Mn_2_^2+ (c)^	Asp_57_-Mn_1_^2+^	Asp_57_-Mn_2_^2+^	Asp_85_-Mn_1_^2+^
1 nM	R (p)	0.794 (0.108)	0.301 (0.623)	−0.171 (0.784)	0.989 ** (0.001)	0.914 * (0.030)	0.955 * (0.012)	0.665 (0.221)	0.622 (0.262)	0.445 (0.452)
10 nM	R (p)	0.735 (0.157)	0.293 (0.633)	−0.017 (0.978)	0.994 ** (0.001)	0.928 * (0.023)	0.904 * (0.035)	0.773 (0.125)	0.549 (0.338)	0.325 (0.593)
100 nM	R (p)	0.660 (0.225)	0.246 (0.690)	0.146 (0.815)	0.964 ** (0.008)	0.886 * (0.046)	0.817 (0.091)	0.858 (0.063)	0.470 (0.424)	0.177 (0.776)
Correlation Analysis Data ^a^		Ionic bond	Active center exposure (Å^2^)							
		Asp_85_-Mn_2_^2+^	Asn_117_ + Mn_1_^2+^	Asp_85_ + Mn_1_^2+^	Asp_57_ + Mn_1_^2+^	His_241_ + Mn_1_^2+^	Asp_85_ + Mn_2_^2+^	Asp_57_ + Mn_2_^2+^		
1 nM	R (p)	−0.171 (0.784)	0.874 (0.053)	−0.955 * (0.011)	−0.787 (0.114)	−0.885 * (0.046)	−0.735 (0.157)	−0.628 (0.257)		
10 nM	R (p)	−0.017 (0.978)	0.819 (0.090)	−0.957 * (0.010)	−0.786 (0.115)	−0.878 (0.050)	−0.759 (0.137)	−0.658 (0.228)		
100 nM	R (p)	0.146 (0.815)	−0.739 (0.154)	−0.809 (0.097)	−0.724 (0.166)	−0.629 (0.255)	−0.724 (0.166)	−0.629 (0.255)		

^a^: Sample size n=15; ^b^: The inhibition effect of MCs at three test concentrations; ^c^: Glu^6^-Mn_2_^2+^ is the interactions between carbonyl O/hydroxyl O of Glu^6^ and Mn_2_^2+^, respectively; logP (o/w) is the Log octanol/water partition coefficient; logS is the water solubility parameter; R is the Pearson correlation between inhibition data and the major interaction parameters; p is the 2-tailed significance of the related data; ** means significant at the 0.01 level; * means significant at the 0.05 level.

## Data Availability

Not applicable.

## Supplementary Materials

The following supporting information can be downloaded at: https://www.mdpi.com/article/10.3390/toxins14060390/s1. All data generated or analyzed during this study are included in this published article and its supplementary information files, Table S1: Main interaction parameters for the complexes of MCs and PP2A.
