# Supplementary material for: Insight into the Molecular Mechanism for the Discrepant Inhibition of Microcystins (MCLR, LA, LF, LW, LY) on Protein Phosphatase 2A
Source: Toxins (Basel). 2022 Jun 3;14(6):390. doi: 10.3390/toxins14060390 (PMC9227578; doi:10.3390/toxins14060390)
Supplement: Supplementary file 1 [file toxins-14-00390-s001.zip › toxins-1744245-supplementary.pdf]

toxins

MDPI

Article

Insight into the Molecular Mechanism for the Discrepant Inhibition of Microcystins (MCLR, LA, LF, LW, LY) on Protein Phosphatase 2A

Yixue Xu, Jiyuan Cui, Huiqun Yu and Wansong Zong

Table S1. Main interaction parameters for the complexes of MCs and PP2A.

Molecular simulation parameters		MCLR	MCLF	MCLA	MCLY	MCLW
Combination energy (KJ/Mol)		-5388.9326	-5338.0981	-5362.9365	-5350.8169	-5360.7036
Combination area (Å^2)	Total	683.0693	650.6934	645.44495	675.6512	691.5637
	Ala^1→PP2A	67.2139	67.2231	67.0766	66.0649	67.3914
	Leu^2→PP2A	145.0408	144.5102	145.8004	145.7317	145.8744
	MeAsp^3→PP2A	71.5026	70.4844	70.3587	70.1468	70.9335
	Z^4→PP2A	102.7079	60.664	60.3979	94.6483	122.0323
	Adda^5→PP2A	369.1675	362.4834	361.8033	361.2742	363.7784
	Glu^6→PP2A	172.7180	167.3475	167.3732	167.1054	168.6401
	Mdha^7→PP2A	118.6913	115.3184	116.166	115.2095	115.5349
logP (o/w)		-77.5350	-75.6480	-75.6830	-76.0820	-76.044
logS		-249.6839	-250.4136	-250.3514	-250.7902	-250.1035
Positive accessible surface area (Å^2)	Total	438.9368	412.5568	409.0258	424.1979	445.4278
	Ala^1→PP2A	35.0459	36.1737	36.2160	35.6512	36.3976
	Leu^2→PP2A	90.8750	91.9079	93.3103	92.1142	93.7569
	MeAsp^3→PP2A	40.0453	40.0493	39.2179	39.7786	40.7106
	Z^4→PP2A	64.9071	41.6399	41.2726	61.1886	86.0338
	Adda^5→PP2A	242.0883	240.7445	239.7152	239.7065	240.7343
	Glu^6→PP2A	97.3617	95.1693	94.1602	94.9192	95.3361
	Mdha^7→PP2A	78.1601	78.6281	78.7967	78.1179	78.4523
Negative accessible surface area (Å^2)	Total	244.13275	238.13685	236.419	241.45355	246.13545
	Ala^1→PP2A	25.1677	31.0497	25.8606	30.414	30.9933
	Leu^2→PP2A	52.1661	52.6026	52.4898	53.6178	52.1173
	MeAsp^3→PP2A	32.4573	31.4351	30.281	32.5184	30.2127
	Z^4→PP2A	37.8009	19.0276	19.1255	33.4602	35.998
	Adda^5→PP2A	124.0795	124.2891	123.6979	123.5169	124.044
	Glu^6→PP2A	73.3566	73.1885	73.6716	73.9164	73.9041
	Mdha^7→PP2A	36.5313	36.6906	36.2891	36.8919	36.2342
Hydrophobic surface area (Å^2)	Total	404.7893	418.4991	412.6355	416.9352	415.4368
	Ala^1→PP2A	16.6175	16.2406	17.3727	16.5160	17.0139
	Leu^2→PP2A	59.2876	58.7177	58.8369	59.1769	58.9443
	MeAsp^3→PP2A	6.6383	7.2477	6.7786	7.5194	7.2099
	Z^4→PP2A	39.8851	46.4391	45.0562	44.7422	43.5330
	Adda^5→PP2A	251.0276	249.5083	248.9231	248.9479	249.8346
	Glu^6→PP2A	52.8923	52.5383	52.6932	52.1723	52.9607
	Mdha^7→PP2A	80.8027	78.5997	77.2210	76.9234	78.0296
Hydrogen bonds (KJ/Mol)	Total	-39.7	-30.2	-27.7	-26.4	-28.2
	Z^4→Pro$_{213}$	-6.5	-3.0	---	---	---
	Ala^1←Arg$_{268}$	-2.1	-2.0	-2.1	-2	-2.2
	Leu^2←Arg$_{89}$	-3.0	-2.1	-2.6	-2.5	-2.5
	MeAsp^3←Arg$_{89}$	-7.4	-5.4	-5.6	-5	-5.9
	Z^4←Arg$_{214}$	-6.4	-4.5	-4.7	-4.3	-4.8

	**Adda[5]←His$_{118}$**	-1.5	-1.8	-1.5	-1.5	-1.5
	**Glu[6]←Arg$_{89}$**	-6.1	-5.2	-5.3	-5	-5.7
	**Mdha[7]←Arg$_{268}$**	-5.8	-5.9	-5.8	-6	-5.1
	**Adda[5]←Asn$_{117}$**	-0.9	-0.3	-0.1	-0.1	-0.5
**Metal bonds (KJ/Mol)**	**Total**	-38.8	-38.7	-38.8	-38.6	-38.8
	**Glu[6]-Mn$_1^{2+}$**	-7.0	-7.1	-7	-7.1	-7.2
	**Glu[6]-Mn$_2^{2+}$**	-9.2	-9.3	-9.3	-9.3	-9.4
	**Asp$_{57}$-Mn$_1^{2+}$**	-4.5	-4.7	-4.7	-4.6	-4.6
	**Asp$_{57}$-Mn$_2^{2+}$**	-5.4	-5.6	-5.7	-5.6	-5.7
	**Asp$_{85}$-Mn$_1^{2+}$**	-5.2	-4.5	-4.5	-4.5	-4.5
	**Asp$_{85}$-Mn$_2^{2+}$**	-4.6	-4.2	-4.3	-4.3	-4.3
	**His$_{241}$-Mn$_1^{2+}$**	-2.7	-3.3	-3.3	-3.2	-3.1
**Ionic bonds (KJ/Mol)**	**Total**	-101.5	-106.2	-116.3	-116.3	-115.8
	**Leu[2]←Arg$_{89}$**	-0.6	-0.8	-0.6	-0.6	-0.7
	**MeAsp[3]←Arg$_{89}$**	-6.9	-6.9	-6.9	-6.9	-6.8
	**Glu[6]←Arg$_{89}$**	-3.6	-7.1	-7.0	-7.2	-6.5
	**Glu[6]-Mn$_1^{2+}$**	-11.9	-12.3	-12.3	-12.2	-12.2
	**Glu[6]-Mn$_2^{2+}$**	-22.5	-23.1	-23.2	-23.2	-23.3
	**Asp$_{57}$-Mn$_1^{2+}$**	-11.6	-11.7	-11.7	-11.7	-11.6
	**Asp$_{57}$-Mn$_2^{2+}$**	-15.3	-15.2	-25.3	-25.3	-25.4
	**Asp$_{85}$-Mn$_1^{2+}$**	-16.7	-16.7	-16.9	-16.8	-17.0
	**Asp$_{85}$-Mn$_2^{2+}$**	-12.4	-12.4	-12.4	-12.4	-12.3
**Active center exposure (Å^2)**	**Asn$_{117}$ + Mn$_1^{2+}$**	322.5904	321.0793	319.6803	320.0182	319.7402
	**Asp$_{85}$ + Mn$_1^{2+}$**	310.286	313.9596	315.4838	315.7891	314.0673
	**Asp$_{57}$ + Mn$_1^{2+}$**	314.8653	318.9475	316.9461	316.9527	317.5759
	**His$_{241}$ + Mn$_1^{2+}$**	352.8933	356.9761	357.7964	356.0291	356.7424
	**Asp$_{85}$ + Mn$_2^{2+}$**	319.806	321.3629	323.0891	321.5287	321.2937
	**Asp$_{57}$ + Mn$_2^{2+}$**	312.4394	315.1101	313.7313	313.2443	313.6917

note: --- no related parameter was detected.
